# Identification of a Novel Binding Partner of Phospholipase Cβ1: Translin-Associated Factor X

**DOI:** 10.1371/journal.pone.0015001

**Published:** 2010-11-29

**Authors:** Omozuanvbo R. Aisiku, Loren W. Runnels, Suzanne Scarlata

**Affiliations:** 1 Department of Physiology and Biophysics, Stony Brook University, Stony Brook, New York, United States of America; 2 Department of Pharmacology, University of Medicine and Dentistry of New Jersey-Robert Wood Johnson Medical School, New Brunswick, New Jersey, United States of America; University of South Florida College of Medicine, United States of America

## Abstract

Mammalian phospholipase Cβ1 (PLCβ1) is activated by the ubiquitous Gα_q_ family of G proteins on the surface of the inner leaflet of plasma membrane where it catalyzes the hydrolysis of phosphatidylinositol 4,5 bisphosphate. In general, PLCβ1 is mainly localized on the cytosolic plasma membrane surface, although a substantial fraction is also found in the cytosol and, under some conditions, in the nucleus. The factors that localize PLCβ1in these other compartments are unknown. Here, we identified a novel binding partner, translin-associated factor X (TRAX). TRAX is a cytosolic protein that can transit into the nucleus. In purified form, PLCβ1 binds strongly to TRAX with an affinity that is only ten-fold weaker than its affinity for its functional partner, Gα_q_. In solution, TRAX has little effect on the membrane association or the catalytic activity of PLCβ1. However, TRAX directly competes with Gα_q_ for PLCβ1 binding, and excess TRAX reverses Gα_q_ activation of PLCβ1. In C6 glia cells, endogenous PLCβ1 and TRAX colocalize in the cytosol and the nucleus, but not on the plasma membrane where TRAX is absent. In Neuro2A cells expressing enhanced yellow and cyano fluorescent proteins (i.e., eYFP- PLCβ1 and eCFP-TRAX), Förster resonance energy transfer (FRET) is observed mostly in the cytosol and a small amount is seen in the nucleus. FRET does not occur at the plasma membrane where TRAX is not found. Our studies show that TRAX, localized in the cytosol and nucleus, competes with plasma-membrane bound Gα_q_ for PLCβ1 binding thus stabilizing PLCβ1 in other cellular compartments.

## Introduction

Inositide-specific mammalian phospholipase Cbeta (PLCβ) enzymes are the main effectors of the Gα_q_ family of G proteins and are coupled to agents such as angiotension, dopamine, serotonin, bradykinin, etc. (for review see [1], [2], [3], [4]). PLCβ catalyzes the hydrolysis of the signaling lipid phosphatidylinositol 4,5 bisphosphate (PI(4,5)P_2_) to produce the second messengers inositol 1,4,5 trisphosphate and diacylglycerol that in turn stimulate the release of Ca^2+^ from intracellular stores and activate protein kinase C, respectively. Both the Gα_q_ family of G proteins and the PLCβ enzymes they activate are found in all mammalian cells lines.

In cultured cells, PLCβ1 resides mainly on the surface of the plasma membrane where it associates with its membrane-bound activator Gα_q_ and can access its PI(4,5)P_2_ substrate. In addition to this plasma membrane population, a significant population of PLCβ1 resides in the cytosol, and under some circumstances, in the nucleus. The factors that localize PLCβ1 to these alternate compartments are unknown, especially since PLCβ1 is expected to have a high propensity to localize to the plasma membrane due to its strong, non-specific lipid binding behavior [5]. Additionally, the basal activity of PLCβ1 is very low and it is unclear how it can be activated in these alternate compartments since Gα_q_ appears to only reside at the plasma membrane (see [6], [7]).

There are several possible mechanisms that may underlie the cytosolic localization of PLCβ1. The first might be a saturation of binding sites on the plasma membrane. While we lack the knowledge to accurately quantify binding sites and the local cellular concentration of competing proteins, we note that the cellular concentration of Gα_q_ appears to be higher than PLCβ1 allowing Gα_q_ to interact with its other effectors, phosphatidylinositol 3-kinase and RhoGEF (see [8]). Another possibility is that one or more cytosolic proteins might promote the plasma member localization of PLCβ1. With this idea in mind, we searched for alternate protein partners of PLCβ1 using a yeast two hybrid approach and identified the protein TRAX (translin-associated factor X). TRAX forms strong complexes with its only known partner, translin [9]. Translin is a single-stranded DNA and RNA-binding protein with proposed functions in chromosomal translocations in lymphoid cells and mRNA transport and storage in brain and testis [10]. Both translin and TRAX are part of the RNA-induced silencing complex (RISC) where they help guide double stranded RNA into the silencing machinery [11]. Additionally, TRAX has been implicated to function as a localization factor for translin. When the cellular level of translin exceeds TRAX, it remains in the cytosol [12]. However, when the cellular level of translin is reduced, translin can partition into the nucleus through the nuclear localization signal (NLS) of TRAX.

Since TRAX appears to regulate the cellular localization of translin, it is possible that TRAX may similarly modulate the localization of other cellular proteins. In this study, we show that TRAX and PLCβ1 interact strongly in solution and form complexes in living cells. We find that TRAX competes with Gα_q_ for PLCβ1 binding and activation. Our studies show that TRAX stabilizes the cytosolic and nuclear localization of PLCβ1.

## Methods

### Sample Preparation

Purified proteins were used in all in vitro experiments. His-tagged PLCβ1 from rat and Gα_q_ from rat were expressed in Sf9 cells and purified based on previously described methods (see [13], [14]). Preparation of C-terminal truncated PLCβ1 has been described [5]. Large, unilamellar vesicles (LUVs), 100nm in diameter, were prepared by extrusion. All lipids (1-palmitoyl-2-oleoyl phosphatidylethanolamine (POPE), 1 -palmitoyl-2-oleoylphosphatidylserine (POPS), 1-palmitoyl-2-oleoylphosphatidylcholine (POPC)) were purchased from Avanti Polar Lipids (Alabaster, AL) with the exception of the tritiated PI(4,5)P_2_ which was purchased from Perkin Elmer. Human TRAX cDNA purchased from Open Biosystems was cloned into pET32a expression vectors purchased from Novagen. His-tagged TRAX was expressed in Rosetta cells and purified on a Ni-NTA column. The integrity of the TRAX preparation was determined by western blot analysis and circular dichroism spectra (see results).

### Protein Labeling and Reconstitution

In vitro binding studies were carried out by labeling either the PLCβ1 or Gα_q_ constructs with the thiol reactive probe 7-diethylamino-3- (4′-maleimidylphenyl)-4-methylcoumarin (CPM, Invitrogen, Inc) as previously described to yield fluorescent proteins with an ∼1∶1 probe:protein ratio (see [15]). We note that labeling by this method does not affect the activity of PLCβ1 or its ability to be stimulated by Gα_q_ (see [14]). To prevent protein aggregation, Gα_q_ was labeled in the presence of storage detergents (0.1% lubrol) and was reconstituted into preformed extruded lipid vesicles by simple addition.

### Optical Measurements and Data Analysis

CD spectra were carried out on an Olis RSM 1000 CD spectrophotometer (On-line Instrument Systems, Inc. Bogart, GA). TRAX concentration was 20 µM in 20 mM Hepes, 0.16 M NaCl, pH 7.4. Binding affinity measurements were performed on an ISS spectrofluorometer (Urbana, IL) using a 3 mm cuvette (see [15]). CPM-labeled proteins were excited at 380 nm and scanned 420 to 560 nm. Binding affinity was determined as a function of the increase in CPM fluorescent intensity as non-fluorescent protein was incrementally added. Briefly, the sample spectra were corrected by subtracting out identical spectra in control cuvettes that substituted buffer for non-fluorescent protein. The area under the emission peaks of the corrected spectra were calculated using ISS Vinci software. These area were then plotted as a function of concentration of added protein, and the resulting curves were fit to a bimolecular dissociation constant using Sigma Plot (Jandel, Inc.).

Membrane binding studies were carried out by measuring the change in intensity of a 20 µM solution of CPM- PLCβ1 as freshly extruded large unilamellar vesicles were incrementally added. The data were corrected for background by carrying out an identical titration where buffer was substituted for CPM- PLCβ1, The corrected data were them fit to a hyperbolic curve using Sigmaplot (Jandel, Inc.).

### Activity Measurements

Measurements were made using full length and truncated His-tagged PLCβ1, Gα_q_, and TRAX and small, unilamellar vesicles consisting of PI(4,5)P_2_, POPE, POPS (1∶1∶1) doped with ^3^H-PI(4,5)P_2_ prepared by sonication (see [13]). Briefly, 2mM of lipid were incubated at 37°C with PLCβ1 in the linear range of the activity as determined for each by running a time course experiment (usually between 2 and 5 minutes). The reaction was initiated by the addition of Ca^2+^. Activities are reported as the percent of radioactive PI(4,5)P_2_ hydrolysis that occurred.

### Cell Culture

Neuro2A cells from American Type Culture Collection (www.ATCC.org) were cultured in 50/50 DMEM/F12 media supplemented 10% FBS. C6 glioma cells were cultured in DMEM containing 10% FBS, 100mM sodium pyruvate and 1% PenStrep at 37° C with 5% CO_2_.

DNA was transfected into cells by electroporation using a protocol adapted from Maniatis. Cells were grown to near 100% confluence and washed with sterile PBS. The cells were then trypsinized, centrifuged 5 minutes at 1500× g and resuspended in 10mL of fresh growth medium. 800 µl of cells were pipeted into a 0.4cm BioRad cuvette and placed in an electroporator (BioRad Gene Pulser Xcell). Cells were then plated and covered with fresh medium.

### Immunofluorescence

Cells were fixed and stained with primary antibodies to TRAX and PLCβ1 (Santa Cruz Biochemicals, Inc.). In certain cases as noted in the text, eYFP-tagged PLCβ1was transfected via electroporation prior to fixing. Cells were initially washed with PBS + 1mM Ca^2+^ and 2 mM Mg^2+^. Cells were then fixed with 3.7% formaldehyde in PBS and permeabilized with a solution of 0.2% NP40 in PBS. After, the cells were blocked in 4% goat serum in 1X TBS, washed and a primary antibody added. Cells were incubated for 1 hour, washed and treated with a secondary antibody. After another wash, the cells were viewed on either a Zeiss Axiovert 200M with an AxioCam MRm camera, or an Olympus Fluoview FV1000 laser confocal microscope. Data were analyzed using either Olympus (Fluoview) software or Image J (NIH).

### FRET analysis

A fully functional eYFP-tagged PLCβ1 was cotransfected with TRAX fused with eCFP. Cells were allowed to incubate for 48 hours. Afterwards the live cells were viewed through the CFP, YFP and FRET channels and FRET measurements on regions of interest were made using sensitized emission on an Olympus Fluoview FV1000 laser confocal microscope (for details, see [16]). Analysis was done using Fluoview software (Olympus, Inc.).

## Results

### Identification of TRAX as a PLCβ1 binding partner

A yeast two-hybrid (Y2H) screen of a rat brain library with the C2 domain-containing COOH-terminus of rat PLCβ1 (a.a. 643-1216) was performed to identify novel interacting partners important to the functional regulation of PLCβ1 and uncovered the TRPM7 ion channel as a binding partner [17], [18]. Among the 69 rat Y2H clones from the screen that were sequenced based on selected growth and β-galactosidase activity, three overlapping clones were identified as translin-associated factor X (TRAX). Since TRAX has a cytosolic and nuclear localization, we carried out further tests to determine whether it is a PLCβ1 binding partner.

**Figure 1:**
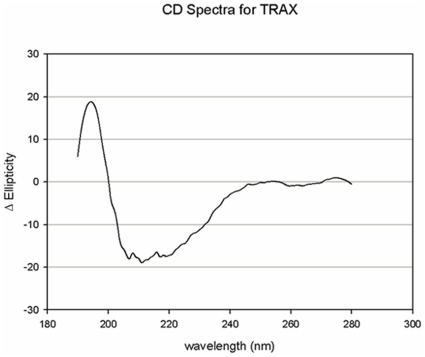
The structure of TRAX is mainly helical. Circular dichroism spectrum of 20 µM TRAX in 20 mM Hepes, 160 mM NaCl, pH 7.4.

Initially, we directly measured the association between purified TRAX and purified PLCβ1 in solution. While the integrity of purified PLCβ1 is readily assessed by its catalytic activity and its ability to be activated by Gα_q_, TRAX does not have a catalytic function. Thus, we determined whether TRAX was folded properly by circular dichroism (CD) spectroscopy. In **Fig. 1** we show the CD spectrum of a solution of 20 µM TRAX in buffer. We find that the TRAX CD spectrum displays a high degree of secondary structure that corresponds to ∼80% helical content (http://www.embl.de/~andrade/k2d). This high helical content is expected from its sequence where it is predicted to have over 50% helical structure with the remainder being loops (www.predictprotein.org).

**Figure 2:**
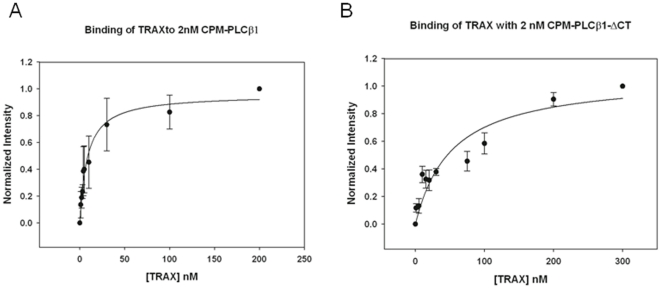
TRAX binds strongly to PLCβ1. **A** – Binding of TRAX to 2 nM CPM- PLCβ1 as monitored by the increase in CPM intensity where the normalized fluorescence intensity is shown as a function of TRAX concentration. In these studies, an 80% increase in intensity was observed as compared to control samples that substituted buffer for TRAX. Also shown is the fitted curve to a bimolecular dissociation constant where *Kd* = 8±1 nM (n = 6 and S.D. is shown). **B** – Identical study as **2A** except that the COOH-terminal deletion mutant of PLCβ1 (PLCβ1-ΔC) was used instead of the full length enzyme (n = 3 and S.D. is shown). While a binding curve is shown to guide the eye, the affinity between the proteins was too weak to be accurately fit to a bimolecular dissociation constant. We note that the total change in CPM intensity was also ∼80% at the end of the titration.

We measured the association between TRAX and PLCβ1 using fluorescence methods. In these experiments His_6_- PLCβ1 was expressed in Sf9 cells and purified, and then labeled with the fluorescent probe CPM (diethylamino-3- (4′-maleimidylphenyl)-4-methylcoumarin). We then monitored changes in the fluorescence spectrum of CPM- PLCβ1 with the addition of purified TRAX (see methods and [15] for details). We find that the fluorescence intensity increased systematically with the addition of TRAX without a significant shift in the emission spectrum. Fitting this increase (an 80% increase relative to control samples that did not contain TRAX) to a bimolecular dissociation curve gives an apparent dissociation constant of *Kd* = 8+/−1 nM (**Fig. 2a**). Interestingly, this apparent *Kd* is only ∼10 fold weaker than the one measured for the interaction between PLCβ1 and activated Gα_q_ [14]. We repeated the titration using an initial concentration of PLCβ1 that is above the *Kd*, 10 nM, to verify that the association was shifted to the stoichiometric regime, and a midpoint of 10 nM was obtained (*data not shown*).

The COOH-terminal region of PLCβ1 was used as bait to identify TRAX in the yeast 2-hybrid screen suggesting that this region binds TRAX. Therefore, we repeated the fluorescence binding study using a COOH-terminal deletion mutant of PLCβ1 (PLCβ1–ΔC). We find that the loss of the C-terminus results in a drastic reduction in its affinity for TRAX (**Fig. 2b**). This result confirms that the primary interaction site between TRAX and PLCβ1 is within C-terminal region. Thus, all further experiments used the full length enzyme.

### TRAX competes with Gα_q_-GTP for binding to PLCβ1

**Figure 3:**
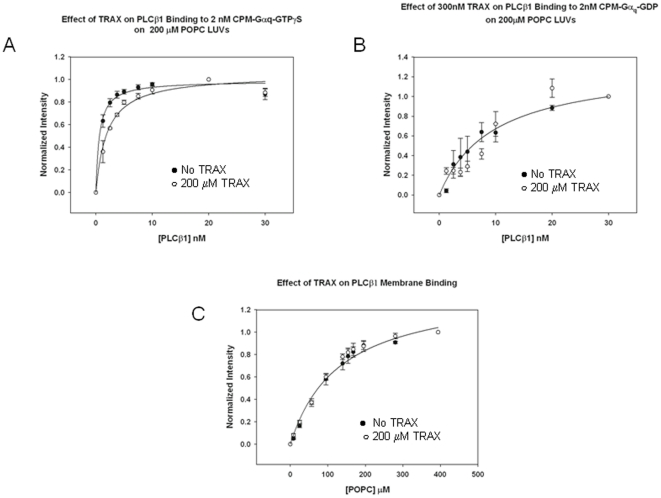
TRAX competes with Gα_q_ for PLCβ1 binding but not membranes. **A** – Binding of PLCβ1 to 2 nM activated CPM- Gα_q_(GTPγS) in the absence (•) and presence (○) of 200 nM TRAX right panel showing the loss in Gα_q_ affinity when TRAX is present, where n = 6 and S.D. is shown. We note that an ∼20% increase in CPM intensity was seen both without and with TRAX. **B-** Binding of PLCβ1 to 2 nM deactivated CPM- Gα_q_(GDP) in the absence (•) and presence (○) of 200 nM TRAX, where n = 3 and S.D. is shown. **C**- Binding of PLCβ1 to PC∶PS∶PE (1∶1∶1) large, unilamellar vesicles in the absence (•) (K_p_ = 132 µM) and presence (○) (K_p_ = 120 µM) of 200 nM TRAX as measured by the increase in CPM intensity as LUVs are titrated into the solution, where n = 3 and S.D. is shown.

PLCβ1 strongly associates with Gα_q_-GTP on membrane surfaces (*Kd* = 0.67 nM) through sites in its C2 and COOH-terminal regions [19], [20]. Since both TRAX and Gα_q_ bind to the COOH-terminal region, we asked whether TRAX would compete with binding of Gα_q_ to full length PLCβ1. For these studies, purified Gα_q_ activated with non-hydrolyzable GTPγS was labeled with CPM and reconstituted onto large unilamellar vesicles (LUVs) composed of POPC∶POPS∶POPE (1∶1∶1). We measured the dissociation constant between PLCβ1 and Gα_q_-GTPγS in the absence and presence of excess TRAX (300nM). The presence of TRAX resulted in a 3-fold reduction in PLCβ1- Gα_q_-GTPγS binding affinity, showing that TRAX competes with Gα_q_ for binding to PLCβ1 (**Fig 3a**).

We repeated the above study substituting unactivated Gα_q_(GDP) for activated Gα_q_ (GTPγS). It is worth noting that in the deactivated state, the affinity between Gα_q_ and PLCβ1 is reduced by a factor of ∼50 [14] giving rise to large experimental error. As expected, we find that TRAX does not appear to compete with deactivated Gα_q_ for binding to PLCβ1. This result may suggest that deactivation of Gα_q_ (GDP) alters its binding interaction with PLCβ1, consistent with previous work [6], to a site that is less competitive for TRAX association (**Fig. 3b**).

PLCβ1 binds strongly to lipid membranes where it associates with Gα_q_ and accesses its substrate. Membrane binding of PLCβ1 has been found to be primarily mediated through the N-terminal PH domain of PLCβ1 and to a lesser extent, its C-terminal region [5]. Since TRAX might affect the association between PLCβ1 and Gα_q_ by altering its membrane binding affinity, we determined whether TRAX would interfere with PLCβ1's association with membranes. We find it does not (**Fig. 3c**). A lack of effect of TRAX on the membrane binding of PLCβ1 correlates well with the finding that TRAX associates with the C-terminal region and not the N-terminal membrane binding region.

### TRAX interferes with the activation of PLCβ1 by Gα_q_ subunits

**Figure 4:**
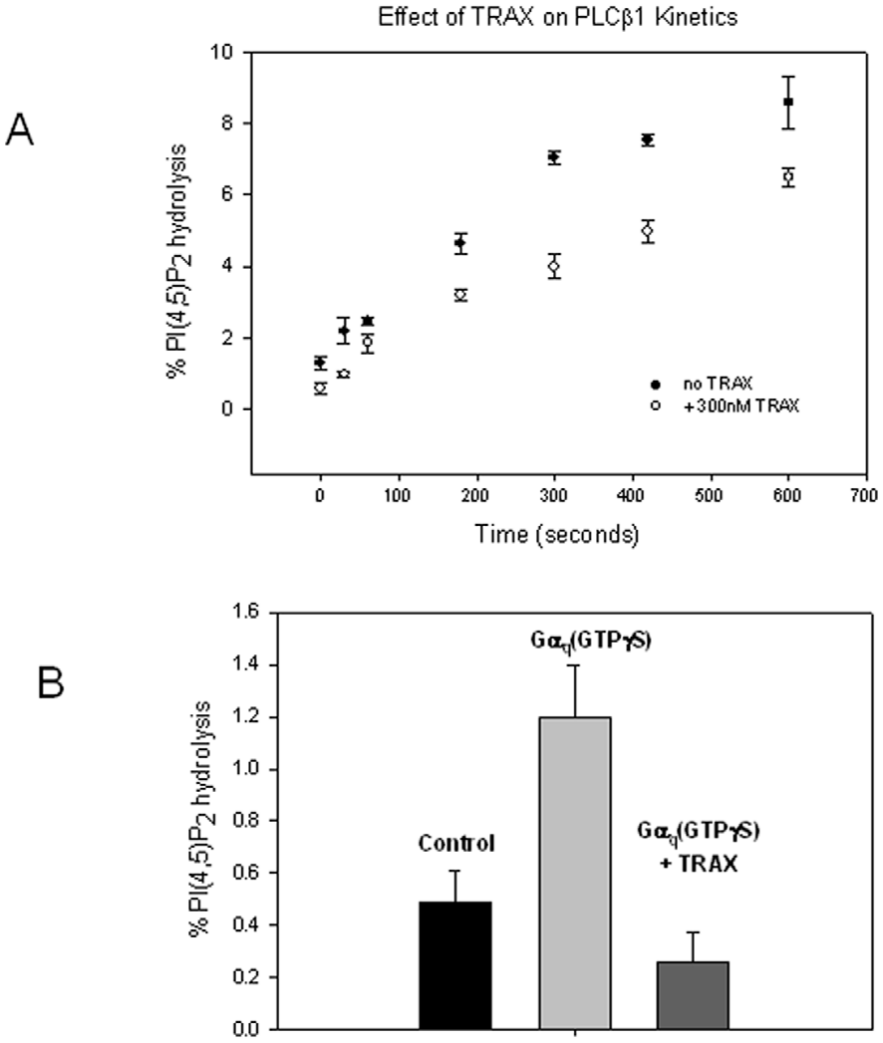
TRAX affects the activation of PLCβ1 by Gα_q_. **A** – The effect of 300 nM TRAX on the rate of PI(4,5)P_2_ hydrolysis catalyzed by 25nM PLCβ1 (n = 3 and S.D. is shown). As can be seen, TRAX does not affect the initial velocity of the curve. **B-** Prevention of activation of 5 nM PLCβ1 by 5nM Gα_q_ by 300 nM TRAX, where n = 8 and S.D. is shown.

We next determined whether TRAX has the ability to modulate the enzymatic activity of PLCβ1 or its activation by Gα_q_. These studies were carried out by monitoring the amount of PI(4,5)P_2_ hydrolysis catalyzed by 2 nM PLCβ1 in the presence and absence of TRAX. We find that a 300 molar excess of TRAX does not greatly affect the initial velocity of the reaction catalyzed by PLCβ1, but reduces the velocity at later times, suggesting that TRAX causes a small reduction in the maximum rate (**Fig. 4a**). We then tested whether TRAX could block PLCβ1 activation by Gα_q_. Again, a large excess of TRAX was used to allow it to compete with Gα_q_, which binds PLCβ1 much more strongly. We find that TRAX prevents the activation of PLCβ1 by Gα_q_ (**Fig.4b**), which is consistent with the ability of TRAX to disrupt the association between PLCβ1 and Gα_q_ (GTPγS) (**Fig.3a**).

### TRAX and PLCβ1 are associated in cultured cells

**Figure 5:**
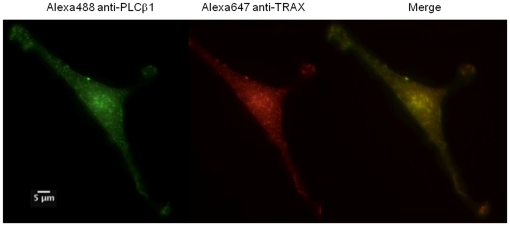
TRAX and PLCβ1 co-localize in C6 glial cells. Example of a co-immunofluorescence study of endogenous PLCβ1 (*left panel*) as visualized by Alexa488-labeled antibody, TRAX (*middle panel*) visualized by Alexa647-labeled antibody and the resulting merged image (*right panel*) in C6 glial cells. The scale bar is 5 µm.

We verified that TRAX and PLCβ1 associate in cells using fluorescence microscopy. First, we used immunofluorescence to determine whether endogenous TRAX and endogenous PLCβ1 are colocalized in C6 glia cells. The images in **Fig. 5** show almost complete colocalization of the proteins throughout the cell suggesting association between the two proteins.

Since colocalization will only indicate whether the proteins reside in the same region of the cell, we measured the physical association of TRAX and PLCβ1 by Förster resonance energy transfer (FRET) using attached “donor” and “acceptor” fluorescent probes. For these studies, we linked enhanced yellow fluorescence proteins (eYFP) to PLCβ1 and enhanced cyano fluorescence protein (eCFP) to TRAX. The observation of FRET from eCYP donors to eYFP acceptors indicates that the probes are at least within 30 Å of each other [21], implying that the two labeled proteins are physically associated.

**Figure 6:**
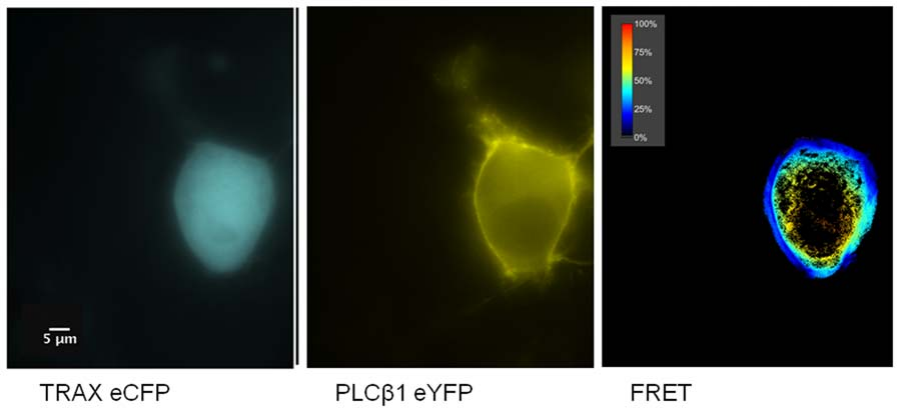
TRAX associates with PLCβ1 in N2A cells. Example of a FRET study showing the raw images of eYFP- PLCβ1 (*left panel*), eCFP-TRAX (*middle panel*) and the normalized FRET image (*right panel*) in transfected Neuro2A cells where amount of FRET is determined by the sensitized emission (see [6]. The scale bar is 5 µm.

We transfected Neuro2A cells with eYFP- PLCβ1 and eCFP-TRAX and measured the amount of FRET by sensitized emission (see methods) using a confocal microscope. FRET values were compared to a positive control consisting of eYFP-X_12_-eCFP and a negative control consisting of free eYFP and eCFP (see [6]). An example of a FRET study is shown in **Fig. 6**. We find that a large degree of FRET occurs between the proteins in the cytosol while only a small amount is seen in the nucleus and none on the plasma membrane compartments since TRAX does not localize to this compartment and cannot provide donor energy for FRET (**Fig. 6**). Averaging the amount of FRET over the entire cell gives a value of 40.1+0.6% (*n = 5*). The lack of FRET in the nucleus contrasts with the coimmunofluorescence results in C6 glia cells and can be explained by a lack of PLCβ1 in the nuclear compartment. Thus, these studies show that TRAX and PLCβ1 associate in the cytosol of cells.

## Discussion

In this study, we have identified TRAX as a novel cellular binding partner of PLCβ1. The potential interaction between PLCβ1 and TRAX was found in a yeast 2-hybrid experiment using the 574 residue C2-domain containing COOH-terminal region of PLCβ1 as bait, and subsequent binding studies between the purified proteins verified this interaction.

The binding of TRAX to the COOH-terminal region of PLCβ1 suggests that this interaction is specific to the PLCβ family rather than the other known mammalian families of PLCs (i.e. δ,γ,ε and ζ) since this region of PLCβ is not found in other PLCs. This region contains the only identified nuclear localization signal of PLCβ1 (a nuclear export signal on PLCβ has never been identified), as well as mitogen-activation protein kinase and protein kinase C phosphorylation sites (we note that unpublished studies by our group suggest that PKC phosphorylation does not affect the interaction between TRAX and PLCβ1: Aisiku, Dowal & Scarlata, *unpublished*). Importantly, the COOH-terminal region of PLCβ1 is necessary for Gα_q_ activation, and has GTPase-promoting activity [19], [22]. Therefore, it is not surprising that TRAX competes with Gα_q_ for binding to PLCβ1. The crystal structure of the isolated COOH-terminal region of PLCβ1 has been solved and is found to be an intertwined helical dimer [23], although it is not clear whether PLCβ1 itself is dimeric. While it is impossible to speculate the mode of interaction between TRAX and PLCβ1, it is notable that translin, TRAX's known binding partner, is comprised of a network of helixes [24].

We assessed the association between endogenous TRAX and PLCβ1 in C6 glial cells, which express these proteins at high levels. We speculate that the high level of expression of PLCβ1 in these cells is responsible for the large cytosolic population since all of the plasma membrane localized Gα_q_ binding sites may be saturated. We find that the two proteins co-localize throughout the cell. We then visualized the associated proteins using FRET by co-expressing fluorescent tagged proteins in a cell line where their endogenous expression is more limited (i.e. Neuro2A cells). We find that the proteins complexes largely reside in the cytosol, since little PLCβ1 localizes to the nucleus and since TRAX is not found on the plasma membrane in these cells. Interestingly, the FRET values that have been reported for PLCβ1 and Gα_q_ [6] are close to the FRET values between PLCβ1 and TRAX observed here. PLCβ1 is known to have a high plasma membrane population due to its strong interaction with Gα_q_ both in the basal and stimulated states [6]. The ability of TRAX to hold PLCβ1 in the cytosol despite the higher affinity of the enzyme for Gα_q_ and the concentrating effect of the membrane in promoting protein association correlates well with the strong association between TRAX and PLCβ1. It is interesting to note that recent biochemical studies suggest that Gα_q_ and PLCβ1 bind to a protein scaffold on the plasma membrane which stabilizes their interactions [25].

While the majority of PLCβ1 resides on the plasma membrane, a significant amount is also seen in the cytosol. Additionally, we can only speculate about the function of PLCβ1 in the cytosol. While its activity is expected to be too low to impact the level of PI(4,5)P_2_ levels in internal membranes, it might be sufficient to keep internal PI(4,5)P_2_ at basal levels.

In C6 glia cells, TRAX and PLCβ1 appear to be co-localized in the nucleus as well as the cytosol. Since both proteins have a nuclear localization sequence, it is just as likely that either partner could be responsible for nuclear transit. Nuclear transit might occur through exposure of either nuclear localization signal upon protein association or upon saturation of PLCβ1's plasma membrane and cytosolic sites. Although PLCβ1 is not considered a nuclear protein, it has been found in the nucleus under some conditions (see [26], [27]). We observe large amount of the enzyme in undifferentiated PC12 cells (Dowal & Scarlata, *unpublished*). Cocco and colleagues find PLCβ1 travels to the nucleus in Swiss 3T3 cells upon stimulation with insulin-like growth factor [28]. The role of PLCβ1 in the nucleus is unknown, but is assumed to be involved in the nuclear phosphadylinositol signaling pathway [29]. It is notable that PLCβ1 does not appear to be associated with the nuclear membrane.

The identification of TRAX as a potential binding partner of PLCβ1 is surprising since TRAX is not known to be associated with inositol phosphate signaling. One study did report a link between TRAX and activation of a G protein coupled receptor linked to cAMP [30] giving rise to the possibility that TRAX may modulate additional aspects of G protein signaling. To date, TRAX has only been known to bind to translin and modulate its cellular localization [12]. It has recently been found that TRAX and translin are part of the RNA-induced silencing complex, and their presence helps to guide RNA insertion into the complex to induce gene silencing [11]. PLCβ1 has been shown to reside in nuclear speckles [29], [31] which are storage deposits for splicing factors and other transcription machinery (for review see [32]). The localization of PLCβ1 in speckles suggests a possible role in RNA splicing and/or in mRNA processing.

While the strong binding constant and large FRET value measured for cytosolic PLCβ1 and TRAX suggest a specific cellular role, the functional consequence of these proteins' association is not yet clear. Unfortunately, we do not yet have the ability to accurately measure the concentrations of these proteins in the cell, but realize that these numbers will change will cell type, localization, cycle and differentiation. Therefore, we cannot determine the importance of TRAX in regulating the localization of PLCβ1 at this time. One possibility is that TRAX buffers PLCβ1 in the cytosol until Gα_q_ becomes activated to high enough levels to displace TRAX from PLCβ1. TRAX may also regulate the entry of PLCβ1 into the nucleus so that it may participate in RNA processing. Alternately, PLCβ1 may regulate entry of TRAX into the nucleus or its association to translin. These functional studies are presently underway.
